# Respect for bioethical principles and human rights in prisons: a systematic review on the state of the art

**DOI:** 10.1186/s12910-024-01049-5

**Published:** 2024-05-22

**Authors:** Massimiliano Esposito, Konrad Szocik, Emanuele Capasso, Mario Chisari, Francesco Sessa, Monica Salerno

**Affiliations:** 1 Faculty of Medicine and Surgery, Kore” University of Enna, 94100 Enna, Italy; 2 Enna, Italia; 3 Interdisciplinary Center for Bioethics, Yale University, New Haven, USA; 4 Department of Social Sciences, University of Information Technology and Management in Rzeszow, Sucharskiego 2 St., 35-225, Rzeszow, Poland; 5 Department of Advanced Biomedical Science-Legal Medicine Section, University of Naples “Federico II”, 80138 Naples, Italy; 6 Department of Medical, Surgical and Advanced Technologies “G.F. Ingrassia”, University of Catania, 95121 Catania, Italy; 7 Catania, Italia

**Keywords:** Bioethics, Human rights, Prison, Torture, Public health, Ethics

## Abstract

**Background:**

Respect for human rights and bioethical principles in prisons is a crucial aspect of society and is proportional to the well-being of the general population. To date, these ethical principles have been lacking in prisons and prisoners are victims of abuse with strong repercussions on their physical and mental health.

**Methods:**

A systematic review was performed, through a MESH of the following words (bioethics) AND (prison), (ethics) AND (prison), (bioethics) AND (jail), (ethics) AND (jail), (bioethics) AND (penitentiary), (ethics) AND (penitentiary), (prison) AND (human rights). Inclusion and exclusion criteria were defined and after PRISMA, 17 articles were included in the systematic review.

**Results:**

Of the 17 articles, most were prevalence studies (n.5) or surveys (n.4), followed by cross-sectional studies (n.3), qualitative studies (n.1), retrospective (n.1) and an explanatory sequential mixed-methods study design (n.1). In most cases, the studies associated bioethics with prisoners’ access to treatment for various pathologies such as vaccinations, tuberculosis, hepatitis, HIV, it was also found that bioethics in prisons was related to the mental health of prisoners, disability, ageing, the condition of women, the risk of suicide or with the request for end-of-life by prisoners. The results showed shortcomings in the system of maintaining bioethical principles and respect for human rights.

**Conclusions:**

Prisoners, in fact, find it difficult to access care, and have an increased risk of suicide and disability. Furthermore, they are often used as improper organ donors and have constrained autonomy that also compromises their willingness to have end-of-life treatments. In conclusion, prison staff (doctors, nurses, warders, managers) must undergo continuous refresher courses to ensure compliance with ethical principles and human rights in prisons.

## Background

The prison system inevitably has ethical repercussions linked to the conditions of the prisoners. First, the detention system does not guarantee privacy or confidentiality. There are also problems inherent in prisoners’ access to treatment, with repercussions on the physical or mental health of prisoners. Furthermore, the ethical repercussions span various sectors. One sector could be that of clinical trials of inmates [1]. Prisoners are often improperly enrolled in clinical trials without adequate informed consent. This raises ethical dilemmas about the voluntariness and conscientiousness of informed consent for recruitment into clinical trials [2]. In fact, as regards the experimentation of offenders in prison, it has always been a bioethical problem. In the USA, during the Second World War, over 400 prisoners were infected with malaria to test the safety and effectiveness of new drugs for the treatment of the disease. Despite the Nuremberg Code, however, in the 1960s and early 1970s prisoners were increasingly exploited. In 1983, in fact, federal rules were issued to regulate research on prisoners and, in some prisons, experimentation on prisoners was prohibited [3].

Another ethical issue in prisons could be the importance of hunger strikes by prisoners, especially against doctors [4]. The doctor, in fact, should clearly inform the striking prisoners about the risks through a multidisciplinary team [5]. However, the doctor often finds himself at a crossroads, namely that of assisting or respecting the will of the prisoner [6]. The detention of women is also an ethical problem. The number of women in prison is increasing, by around 50% compared to 2000. This has ethical implications, as women have special health needs related to specific healthcare approaches, sexual and reproductive health needs, and the treatment of infectious diseases, but also pregnancy and childbirth, caring for children inside and outside prison [7].

Another ethical problem emerged during the COVID-19 pandemic, in which the security of the prison system and the guarantee of human rights were lacking [8]. As claimed by some Authors [9, 10], when the related COVID-19 pandemic arose, some security problems related to the penitentiary system arose. One of these is the balance between security needs and the prisoner’s right to health.

Indeed, a central concept of prisons should be objectives such as guaranteeing the rights of human dignity, rehabilitation, mental health treatment, and freedom from torture or other cruel treatment or punishment [11]. As a recent systematic review shows, healthcare personnel also play a crucial role in enforcing ethical principles within prisons and must have specific training. Correctional nurses should be trained in specific areas such as mental health, drug abuse, emergencies, primary healthcare and public health [12, 13].

This systematic review analyzes the main bioethical implications regarding the prison system, especially with regard to the main topics. To date, the bioethical-prison correlation plays a key role in society since it plays a crucial role in the re-education of inmates to re-enter society. Prisoners cannot be a stigma of society but must be reintegrated and human rights must always be guaranteed in jails.

## Methods

A systematic review was conducted, according to the latest update of the PRISMA statement [14]. Furthermore, Rayyan (http://rayyan.qcri.org), a free web and mobile app, which helped with the initial screening of abstracts and titles, was used independently between authors [15], PubMed, Scopus, and Web of Science (WOS) were used as search engines from 1 January 1950 to 1 January 2024 to evaluate the association between the detention regime and respect for the bioethics of prisoners. The following keywords were used: (bioethics) AND (prison), (ethics) AND (prison), (bioethics) AND (jail), (ethics) AND (jail), (bioethics) AND (penitentiary), (ethics) AND (penitentiary), and (prison) AND (human right). The word “detainees” was not used, since the results obtained with this keyword were limited.

### Inclusion and exclusion criteria

The following exclusion criteria were used: (1) articles not in English, (2) conference papers, (3) reviews, (4) books, (5) conference reviews, (6) editorials and (7) notes. The inclusion criteria were as follows: (1) articles in English, (2) original articles; (3) surveys, (4) longitudinal studies, (5) prevalence studies, (6) cross-sectional studies, (7) retrospective studies, (8) and sequential explanatory mixed-methods study designs.

### Quality assessment and data extraction

M.E. and F.S. initially evaluated all articles, evaluating the title, abstract, and full text. K.S. then reanalyzed the selected articles independently. In cases where there were conflicting opinions on the articles, they were re-evaluated by M.S.

### Characteristics of eligible studies

**Fig. 1:**
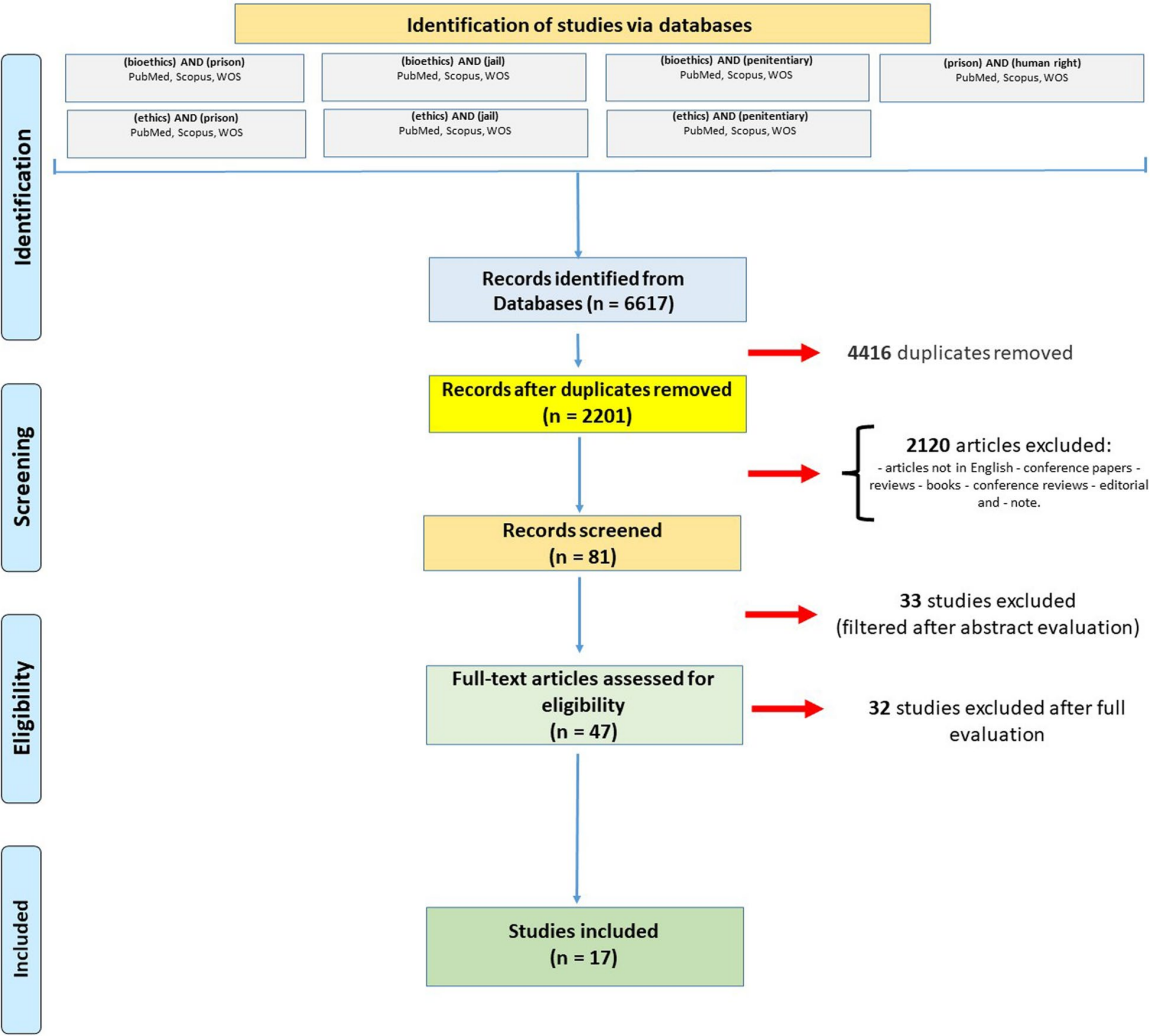
Flow diagram illustrating included and excluded studies in this systematic review

A total of 6617 articles were collected. Of these, 4416 duplicates were removed. Of the 2201 remaining articles, 2120 were removed due to exclusion criteria. Thirty-three studies were excluded after filtering for abstract evaluation. Forty-seven articles were read in full and were assessed for eligibility. Ultimately, 17 articles were included (Fig. 1).

## Results

Seventeen articles were included from the present systematic review. Although all articles on search engines (PubMed, Scopus, WOS) were analyzed regardless of publication date, the articles included were within a time range from 2013 to 2022. This is because, before these years, they were no original articles published, but mostly literature reviews or editorials. Of the 17 articles, in most cases they were prevalence studies (n.5) or surveys (n.4), followed by cross-sectional studies (n.3), finally qualitative studies (n.1), retrospective studies (n.1), and sequential explanatory mixed-methods study designs (n.1). In most cases, the studies associated bioethics with prisoners’ access to treatment for various pathologies such as vaccinations, tuberculosis, hepatitis, HIV, in another bioethics in prisons was correlated with the mental health of prisoners, with disability, with aging, with the condition of women, or with the risk of suicide or the request of prisoners for end-of-life.

Beijersbergen et al. [16] demonstrated that behaving ethically with prisoners was a predictive factor not only of prison order, but also of their psychological well-being. Other studies, such as the one conducted by Cook Earl Prison et al. [17] showed how justice reform during the COVID-19 pandemic could improve the human rights of prisoners. Other studies highlighted how access to care, screening and treatment paths were more difficult within prisons, highlighting a lack of attention to human rights in prisons [18–20]. Finally, this systematic review showed an ethical problem regarding the mental health of prisoners, the condition of women in prisons, and even the end-of-life request for some prisoners with terminal illnesses [21–28]. As for the country where the study was conducted, in most cases it concerned the USA, followed by Europe and, finally, Africa, and then South America.

Table 1 summarizes all articles included in this systematic review.

**Table 1:** Summary of the details of the systematic review

**References**	**Country of the study**	**Kind of study**	**Topic**	**Main findings**
Beijersbergen et al. [16]	Netherlands	Longitudinal study	Mental health and bioethics in prisons	Fair and respectful treatment of prisoners is a predictive factor not only of prison order and respect for rules by prisoners, but also of their psychological well-being
Reinhart et al. [29]	Illinois (Chicago)	Longitudinal study	Pandemic and bioethics in prisons	Study conducted in Cook County Jail on how a new justice reform during the COVID-19 pandemic could improve the human rights of prisoners
Puglisi et al. [18]	Connecticut	Sequential explanatory mixed-methods study design	Cancer incidence and bioethics in prisons	Incarceration is associated with decreased cancer screening rates and a higher risk of hospitalization and cancer death after release from prison. Furthermore, there was evidence of differences between socio-economic status, race and ethnicity during the detention regime
Sasso et al. [19]	Italy	Qualitative descriptive study	Health treatment and bioethics in prisons	An analysis conducted on 31 penitentiary nurses in seven prisons in northern Italy stated that the means of restraint of prisoners does not allow nurses to establish an adequate therapeutic relationship with their patients, giving rise to ethical questions
Elger et al. [20]	Switzerland	Prevalence study	Tuberculosis screening and bioethics in prisons	Prisons have an extremely high prevalence of tuberculosis, multidrug-resistant and extensively drug-resistant tuberculosis and poor treatment outcomes. However, prisoners should have the human right to access at least the same level of tuberculosis care as that offered in their communities
Alemayehu et al. [21]	Ethiopia	Cross-sectional study	Mental health and bioethics in prisons	An incidence of depression was found among prisoners, especially among those with poor general health, long years of imprisonment and worries about children were the most vulnerable. It is absolutely necessary to strengthen mental health services in prisons
Blue et al. [22]	North Carolina	Survey	HIV treatment/prevention and bioethics in prisons	The analysis highlighted a worsening of access to HIV treatment in prison, a negative impact of prison detention on the continuity of HIV treatment, a reduction in privacy and stigmatization. More outside resources are needed, such as from state and local health departments, so that prisons can promptly provide HIV medications to people with disabilities incarcerated in their facilities
Green et al. [23]	Ontario	Survey	Access to care in prison and bioethics in prisons	Usually, people entering prison have a need to access primary care, as in most cases they have unmet health needs. Prison could be a time to guarantee care for these people
Dogbe et al. [24]	Ghana	Survey	Disability in prisons and bioethics	Ninety-nine subjects with disabilities in detention were interviewed; the most common disability was physical, followed by visual, auditory, linguistic, mental and albinism. The study demonstrated the great difficulties these people encountered during their detention regime in Ghana
Shaw et al. [25]	Switzerland	Survey	Assisted suicide in prison and bioethics	Six inmates were interested in assisted suicide while in prison. Some inmates seek assisted suicide for medical reasons, others because they believe it is undignified to spend the rest of their lives in prison. However, there are strong ethical debates about this opportunity
Wangmo et al. [26]	Switzerland	Retrospective study	Aging in prisons and bioethics	For older age groups, more healthcare needs were required in prisons. The poorer health conditions of the elderly, their higher healthcare burden posed an ethical debate on the provision of healthcare for inmates aging in prison
Jones et al. [27]	Ontario	Cross-sectional study	Women's mental health in prisons and bioethics	The female population requiring intensive psychiatric care in an Ontario prison was 3.4%, the ethical issue on the worsening of female psychiatric pathology is still debated
Shrestha et al. [30]	Nepal	Cross-sectional study	Risk of suicide and depression in prisons and bioethics	Approximately 2.3% reported suicidal ideation while incarcerated and 0.9% attempted suicide within prison. It was significantly associated with prior incarceration, poor self-rated health, and weight loss. The ethical issue is always debated and a major social problem
Strodel et al. [31]	Washington, D.C	Prevalence study	COVID-19 vaccination in prisons and bioethics	During the vaccination campaign against COVID-19 it was shown that prisoners had difficult access to vaccines, highlighting an ethical problem for prisoners in treatment
de Araújo et al. [32]	Brazil	Prevalence study	Women in prisons and bioethics	In Brazil, 39% of women with children in prison had children under the age of 10 who were then entrusted to the care of others. Prisons were crowded, with more than 2/3 of inmates sharing a cell with 6 or more inmates. Women had not been screened for cervical or breast cancer in the past 3 years
Crowley et al. [33]	Ireland	Prevalence study	HCV treatment in prisons and bioethics	A major barrier to HCV care and treatment in prisons was highlighted. Incarceration could provide a unique opportunity to enhance HCV treatment and strengthen community connections
Liu et al. [34]	Northern California	Prevalence study	COVID-19 treatment in prison and bioethics	Prisoners also revealed insufficient access to masks, which was associated with an increase in COVID-19 cases and worsening mental health. Prison settings present significant challenges in maintaining infection control and human rights

## Discussion

One of the basic concepts of the detention system in developed countries is to consider prison as the place where criminals are imprisoned as punishment and not for punishment. This concept, although banal, hides an important reflection since it justifies a system in which the prison administration does not want or cannot guarantee dignified detention conditions [35]. Furthermore, it also facilitates the establishment of a repressive prison system, especially towards prisoners who "deserve" such punishment, due to the serious crimes for which they have been convicted. This concept deserves ethical reflection, if it is necessary to treat prisoners differently based on their crimes. In reality, the detention system should be the same for all prisoners regardless of the crime for which they were convicted [36]. Another ethical issue in prisons could be the importance of hunger strikes by prisoners. According to some Authors, the ethical aspects of the doctor during the hunger strikes organized by prisoners are debated and difficult to understand. As defined by the International Committee of of the Red Cross, doctors should intervene by feeding the patient only if the hunger strike is not intentional and conscious but due to a mental pathology (depression, dementia). In case the hunger strike is conscious, the doctor should comply with the prisoner’s wishes [37]. However, Caenazzo et al. [38] correctly highlight that, sometimes, the Courts transfer the convicted person to hospital to be fed compulsorily by health workers. The essential role of the doctor and the ethical consultant in any case remains that of communication, helping them to understand the importance of their gesture.

Prisoners are at enormously greater risk of intentional self-harm and suicide than the general population [39]. An Australian study shows that nearly half of adult prisoners reported lifetime attempted suicide ideation [40]. A dissatisfaction with prisoners’ mental health care is one reason [41]. In 2015 the United Nations issued the Standard Minimum Rules for the Treatment of Prisoners (the "Nelson Mandela Rules") according to which prisoners are entitled to a standard of healthcare at least equivalent to that available outside prison [42]. Furthermore, violence between prisoners is also common and this could lead to an exacerbation of symptoms of mental illness. Restrictive practices, such as periods of solitary confinement, also increase the risk of suicide in prison.

In the present systematic review, the study by Shrestha et al. [30] highlighted that approximately 2.3% of prisoners reported suicidal ideation during detention and 0.9% attempted suicide in prison. The risk of suicide was significantly associated with prior incarceration, poor self-rated health, and weight loss. The authors underlined that the ethical issue was always debated and is a serious social problem. Shaw et al. [25], however, underlined a great ethical dilemma, namely the possibility of leading some prisons that request it to commit assisted suicide. Some prisoners, in fact, ask for assisted suicide for medical reasons, others because they believe it is not dignified to spend the rest of their lives in prison. However, there are strong ethical debates about this opportunity.

For example, Della Croce [43] supports the idea according to which the right of access to assisted suicide has to be understood as a freedom that cannot be taken away from detained individuals, since it would mean leaving the State to decide when and how to end the lives of prisoners.

However, this last topic, assisted suicide in prison, is an important ethical element that has yet to find a common agreement [43, 44].

A recent systematic review analyzed the conditions of women with children in prisons in sub-Saharan Africa by evaluating four main themes, including the physical environment of the prison, nutrition, provision of basic necessities, and availability and accessibility of healthcare services for incarcerated children [45]. The study highlighted that there was a serious lack of basic necessities, from inadequate hygiene, sanitation to safe drinking water, exposure to diseases in overcrowded cells, inadequate nutrition, lack of clothing and bedding, and difficulty in accessing pediatric care. This also had obvious repercussions on children’s health, with serious damage to human rights. Self-harm is very common among incarcerated women, too. An estimate conducted in 2016 showed that there were approximately 7,657 incidents of self-harm in prisons, an increase of 4% compared to the previous year. The most common methods of self-harm in women’s prisons consisted of cuts and scratches followed by self-strangulation [46].

In the present study, there were two articles included in the systemic review that confirmed an ethical problem of the female population in prisons. Jones et al. [27], clarified that there was an important ethical debate on the worsening of female psychiatric pathologies that is still debated. de Araújo et al. [32], confirmed that in Brazil, 39% of women with children in prison had children under the age of 10 who were then entrusted to the care of others. Prisons were crowded, with more than 2/3 of inmates sharing a cell with 6 or more inmates. The women had not been screened for cervical or breast cancer in the past 3 years.

Even during the COVID-19 pandemic, several ethical concerns were observed regarding the care and treatment of prisoners in relation to symptoms associated with SARS-CoV-2 [8, 47–49]. A cross-sectional study conducted in a juvenile prison in Portugal demonstrated that during the COVID-19 pandemic a state of anxiety and fear related to the pandemic was implemented in this population that is considered more fragile [50].

A higher prevalence of infection in prison compared to the general population, a delay in vaccination, and a reduction in hospitalization were highlighted, raising ethical questions on the accessibility of prisoner care during the pandemic. A recent systematic review of the literature has also highlighted this problem, proposing prevention strategies within jails [51]. Moreover, the problem does not only concern prisoners but also migrants in hotspots [52].

Forrester et al. [53], also highlighted numerous ethical issues in prisons during the COVID-19 pandemic due to the increased rate of infection, hospitalizations, and mortality from this infection.

However, the management of COVID-19 in Italian prisons aroused considerable concern at the beginning of the pandemic due to the numerous riots that resulted in the death of inmates, damage and escapes.

These data are consistent with those that emerged in the present systematic review, in which during the vaccination campaign against COVID-19 it was demonstrated that prisoners had difficulty accessing vaccines, highlighting an ethical problem for prisoners in treatment, indicating the need for a new justice reform [17, 31].

In the present systematic review, regarding the country where the study was conducted, in most cases it concerned the USA, followed by Europe and, finally, Africa, and South America. These data are important as they highlight that raising awareness of bioethics in prisons, concerns, in most cases, only some continents (Africa, Europe, followed by Africa), while it is a little discussed topic in some states/continents such as central/south America, Asia (China, Japan, Pakistan etc.) or Australia, and Russia. This means that the issue of bioethics in prisons still needs to make important steps, starting from global awareness.

An important bioethical aspect is the transplantation of organs from prisoners sentenced to death. In fact, due to the low percentage of donated organs, since 1984 China has required that those sentenced to death be subjected to organ transplants. However, this resulted in an important ethical dilemma regarding the importance of informed consent in these situations [54]. An important milestone in prison bioethics was reached on January 1, 2015, when Huang Jiefu, director of the China Organ Donation and Transplant Committee and former vice minister of the Ministry of Health, decreed the end of organ transplants from prisoners sentenced to death [55]. On the topic of organ donations from death-row inmates, Lin et al., state that prisoners are subject to conditions of physical and psychological stress that undoubtedly influence the decisions they make and this is often cited by bioethicists as a reason to avoid the use of prisoners executed as organ donors. Furthermore, the American Society of Transplant Surgeons states that the use of organs from executed prisoners is incorrect as it would violate the fundamental principles of transplantation, such as the need for free and voluntary organ donation [56]. Moreover, Santiago-Delpin et al. [57] state that organ transplantation in prisoners raises numerous bioethical questions. First of all, the informed consent expressly declared by the prisoners and their ability to self-determine.

Isailă et al. [58], however, highlighted another ethical aspect of prisons, namely the difficulty of prisoners in reporting aspects of malpractice claims in prisons. This is a medical-legal aspect of great interest and very original, as there are few complaints from prisoners regarding potential damage from malpractice. This is most likely due not only to the prisoner’s lack of awareness but also to the inmate’s difficulty in accessing this type of justice.

From the results of this systematic review that the conditions of prisoners are still an ethical dilemma on several aspects which, despite the ethical/deontological evolution of society, are struggling to progress. This study has highlighted important gaps in the prison system from the point of view of respect for ethics and human rights, especially with regards to informed consent, palliative treatment, end-of-life, the COVID-19 pandemic, and women’s health.

In order to solve this problem, continuous training, the development of continuous support programs and the development of specific skills are essential for prison staff (guards, doctors, administrative staff), especially to address the challenges and requests of the complex situations that emerge in prisons [59, 60]. Some Authors [61] argue that a good prison social climate also improves the results obtainable from the rehabilitation of prisoners. Finally, health campaigns within prisons can also offer an important contribution in improving the social conditions of inmates [62].

## Limitations and strengths of the study

This systematic review has strengths and limitations. As regards the limitations, a first is certainly the low number of articles included (n.17), this is due to the fact that the prisoner population is not a group in which the subjects are subjected to longitudinal studies. A second limitation is the lack of studies tailored to countries with a similar punishment system. Strengths are the number of keywords inserted, 3 databases used and cross-referenced with each other (Pubmed, WOS, Scopus), the independent screening by co-authors in the choice of articles, and, finally, the lack of systematic reviews on this topic. This study is, in fact, the first systematic review on bioethics in prisons.

## Conclusion

The ethical question of prisons plays a crucial role nowadays and is directly proportional to the cultural progress of a society. Considering that in prison, death can also be seen as an escape route to put an end to a sentence that also involves physical and psychological pain and suffering. In prisons, care and support for prisoners is insufficient and prisoners’ needs are often unmet [63, 64]. In some cases, the quality of healthcare was below an acceptable threshold and the courts intervened to restore a standard of care. In California, in fact, the Court ordered a renovation of the healthcare system costing millions of dollars [65, 66]. Episodes of torture and physical violence are very frequent in prisons, with frequent and important neurological/psychiatric sequelae of prisoners [67, 68]. This systematic review has clearly highlighted the critical points of respect for bioethics in the prison system. Furthermore, from this article it emerged that raising awareness of bioethics in prisons is not a global issue, but would seem to concern a few individual nations, demonstrating that global awareness concerning this issue is necessary and crucial.

## Data Availability

Not applicable.
